# Mechanisms of Stage-Transcending Protection Following Immunization of Mice with Late Liver Stage-Arresting Genetically Attenuated Malaria Parasites

**DOI:** 10.1371/journal.ppat.1004855

**Published:** 2015-05-14

**Authors:** Brandon K. Sack, Gladys J. Keitany, Ashley M. Vaughan, Jessica L. Miller, Ruobing Wang, Stefan H. I. Kappe

**Affiliations:** 1 Seattle Biomedical Research Institute, Seattle, Washington, United States of America; 2 Department of Immunology, University of Washington, Seattle, Washington, United States of America; 3 Department of Global Health, University of Washington, Seattle, Washington, United States of America; Case Western Reserve University, UNITED STATES

## Abstract

Malaria, caused by *Plasmodium* parasite infection, continues to be one of the leading causes of worldwide morbidity and mortality. Development of an effective vaccine has been encumbered by the complex life cycle of the parasite that has distinct pre-erythrocytic and erythrocytic stages of infection in the mammalian host. Historically, malaria vaccine development efforts have targeted each stage in isolation. An ideal vaccine, however, would target multiple life cycle stages with multiple arms of the immune system and be capable of eliminating initial infection in the liver, the subsequent blood stage infection, and would prevent further parasite transmission. We have previously shown that immunization of mice with *Plasmodium yoelii* genetically attenuated parasites (GAP) that arrest late in liver stage development elicits stage-transcending protection against both a sporozoite challenge and a direct blood stage challenge. Here, we show that this immunization strategy engenders both T- and B-cell responses that are essential for stage-transcending protection, but the relative importance of each is determined by the host genetic background. Furthermore, potent anti-blood stage antibodies elicited after GAP immunization rely heavily on F_C_-mediated functions including complement fixation and F_C_ receptor binding. These protective antibodies recognize the merozoite surface but do not appear to recognize the immunodominant merozoite surface protein-1. The antigen(s) targeted by stage-transcending immunity are present in both the late liver stages and blood stage parasites. The data clearly show that GAP-engendered protective immune responses can target shared antigens of pre-erythrocytic and erythrocytic parasite life cycle stages. As such, this model constitutes a powerful tool to identify novel, protective and stage-transcending T and B cell targets for incorporation into a multi-stage subunit vaccine.

## Author Summary

Malaria is arguably one of the deadliest infectious diseases in human history. Today, it infects nearly 300 million people each year and kills up to 1 million of those—mostly women and children under the age of 5—and no effective malaria vaccine has been developed. Traditional subunit vaccines for pathogens work by training the immune system to recognize a single pathogen target. Attempts at developing a subunit malaria vaccine have, however, been stymied by the complexity of the parasite genome which encodes a complex life cycle with specific stages in the mosquito, as well as in the liver and blood of the mammalian host. Only the blood stage parasites cause malaria symptoms and mortality. Previously, it was assumed that immunity to malaria is stage-specific, either targeting parasites in the liver or in blood, but not both. The herein described vaccination approach uses genetically engineered, attenuated rodent malaria parasites that are able to infect the mouse liver and replicate, but die shortly before red blood-infectious parasite stages are formed and released. Immunization with these attenuated parasites induces the immune system to build defenses against both parasite stages in the liver and blood. Protection is mediated by multiple arms of the immune system. The antibody arm recognizes parasite targets shared between liver stages and blood stages. This not only demonstrates the optimal potency of this live-attenuated vaccination strategy, but also provides a potential source of new malaria subunit vaccine targets.

## Introduction

Unlike other infectious diseases, malaria parasites continue to defy the development of a protective vaccine. One main difference between pathogens currently amenable to vaccination and malaria parasites is the degree of complexity of the parasites causing malaria, *Plasmodium spp*. These eukaryotic parasites have complex genomes that control elaborate life cycles. They progress through multiple, antigenically distinct stages of replication and infection within mammalian hosts and mosquito vectors—making it difficult to target with traditional vaccination methods[1]. Infection is initiated when a parasitized *Anopheles* mosquito injects tens to hundreds of sporozoites into the dermis of the host. Sporozoites traverse through multiple host cell types in the dermis for minutes to hours until they traverse the vascular endothelium and into the circulation. The sporozoites are then carried into the sinusoids of the liver where they again traverse multiple cell types to reach and infect hepatocytes. This begins the clinically silent liver stage development of infection, during which each parasite undergoes many rounds of replication in a single hepatocyte and eventually forms tens of thousands of red blood cell-infectious exoerythrocytic merozoites. They are released in to the circulation and begin the asexual blood stage (BS) cycle whereby cyclic infection, replication within and lytic release from red blood cells (RBCs) occurs. This rapidly propagates the parasite and causes all malaria-associated morbidity and mortality as parasite numbers expand into the billions. A fraction of parasites terminally develop into gametocytes, which can be transmitted back to a mosquito during blood meal acquisition. To date, malaria vaccination strategies have largely focused on either the sporozoite and liver stages (“pre-erythrocytic”, PE) or BS of infection by targeting parasite antigens specific to each stage[2]. However, success has been limited with these stage-specific approaches, raising the question as to whether there should be a greater emphasis on multi-stage vaccination approaches.

PE vaccines have the advantage of targeting a bottleneck in the parasite population with only tens to a few hundred sporozoites injected in the skin and even fewer successfully infecting the liver. In addition, PE infection is clinically silent and completely eliminating PE parasites (termed “sterile protection”) would prevent BS infection and thus both disease and transmission. Both humoral and cellular immune defenses can contribute to PE immunity. Antibodies against sporozoites can act in the skin to immobilize the parasite and can bind to sporozoites in circulation to prevent hepatocyte infection[3–7]. Once parasites are within hepatocytes, CD8 T cells can target the infected hepatocyte and kill it[8]. However, successful infection of the liver by even a single parasite can lead to fulminant BS infection. Indeed, the stringent requirement for both antibodies and T cells to eliminate 100% of PE parasites has contributed to the limited success of PE subunit vaccine candidates in clinical trials. The first malaria vaccine candidate to reach phase III clinical trials is RTS,S, which targets only the circumsporozoite protein (CSP). RTS,s is capable of significantly reducing the cases of severe disease, but the long-term efficacy of RTS,S in eliciting protection is limited[9,10]. An alternative strategy for PE vaccination is immunization with live-attenuated sporozoites. Radiation-attenuated sporozoites (RAS) and sporozoites administered under chloroquine cover (known as “infection treatment immunization”, ITI) can confer 100% sterilizing PE protection in humans[11–13] Similarly, immunization with genetically attenuated parasites (GAP) has been shown to elicit complete sterile protection against PE infection in mice[14–16]. CD8 T cells are required for live-attenuated sporozoite protection in mice and also correlate with protection in non-human primates[17–22]. However, immunization with RAS requires the development of very large numbers of sporozoite-specific CD8 T cells for complete protection which in animal models needs to account for ~1% of the total CD8 repertoire[23]. While antibodies elicited by attenuated whole parasites are also able to strongly reduce liver infection, they have not been shown to be essential for protection[4,5,17,19,21,24,25].

The parasite BS have been the other major focus of malaria vaccine development efforts. However, attempts to create BS subunit vaccines have been stymied by suboptimal clinical performance perhaps due to the large degree of antigenic variation and polymorphisms within BS proteins and the high parasite burden as compared to the PE stages[26],[27].

In contrast to the stage-specific approaches to malaria vaccine development, targeting PE and BS life cycle stages simultaneously may be more fruitful as PE immunity can reduce the number of developing liver stages, which in turn reduces the number of merozoites released from the liver—thus potentially making BS infection easier to control and eliminate by an immune response. However, there is scant evidence that such stage-transcending protection (STP) is possible and the antigens and immunological mechanisms potentially capable of mediating STP remain undefined. Thus, establishing an STP model and understanding the mechanisms required for potent immunity against multiple parasite stages could be critical in developing fully protective, multi-stage subunit malaria vaccines.

Our previous work has indicated that late liver stage-arresting GAP confer STP[28]. Herein, we build upon this evidence to show that STP can be mediated by both T cells and by antibodies. Furthermore, protective antibodies predominantly rely on F_C_-mediated effector mechanisms and recognize potentially novel protective antigens shared between the late liver stages and BS parasites. Not only do our findings provide a rationale for the development of a late liver stage-arresting GAP as a vaccine candidate, but they also offer a platform to identify novel antigens and investigate the immune mechanisms mediating robust STP against PE stages and BS of *Plasmodium*.

## Results

### Immunization with a late liver stage-arresting GAP confers stage-transcending protection by eliciting cellular and humoral immune responses

*P*. *yoelii (Py) fabb/f—*parasites that are deficient in endogenous fatty acid biosynthesis undergo substantial liver stage growth, develop into late stage exoerythrocytic schizonts but fail to complete differentiation into exoerythrocytic merozoites[29] As a consequence, mice immunized with *Pyfabb/f—*parasites only experience PE infection and are not exposed to BS parasites. As such, they constitute late liver stage-arresting GAP (LAGAP). Mice immunized with LAGAP are not only protected against sporozoite challenge but are also protected against direct intravenous challenge with *Py*-infected RBCs (iRBCs)[28]. However, the immune mechanisms elicited by PE LAGAP-immunization that control and eliminate BS infection remain to be elucidated.

**Fig 1:**
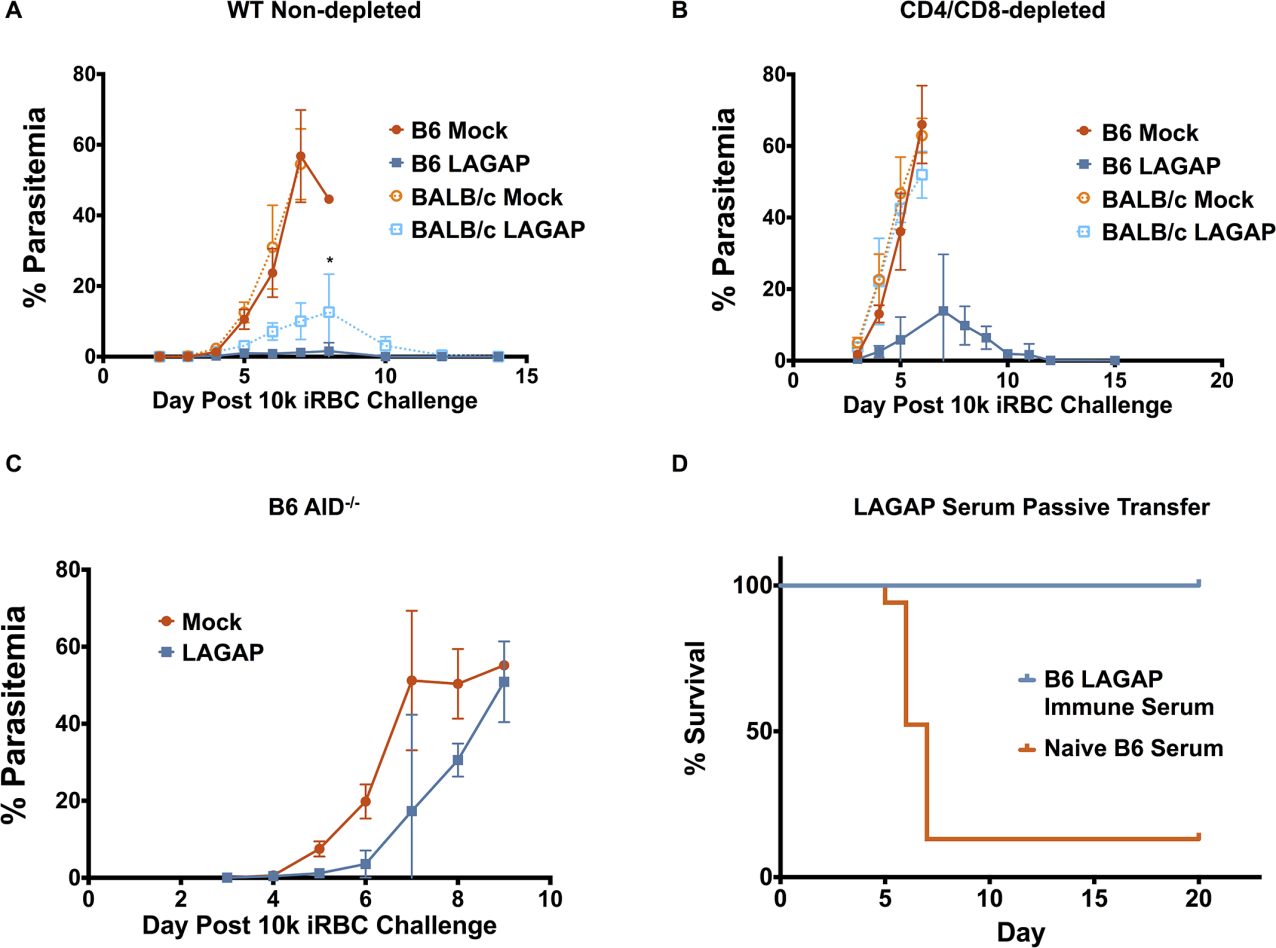
LAGAP immunization elicits T cells and antibodies that can protect against blood stage infection. A) Blood stage parasitemia of BALB/cJ or C57BL/6 mice (n = 5 mice/group) immunized with 3 x 50,000 *Pyfabb/f* ^*-*^ (LAGAP) sporozoites and challenged 3 weeks later with 10^4^ infected red blood cells (iRBC) of a lethal *Py* strain. Both mouse strains show stage-transcending protection (STP). B) Mice (n = 5–9 mice/group over 2 independent experiments) were immunized as in (A) but depleted of CD4 and CD8 T cells using monoclonal antibodies (mAb) 24 hours prior to iRBC challenge. BALB/cJ mice lose STP in the absence of T cells but C57BL/6 mice do not. C) Parasitemia of AID^*-/-*^ mice on the C57BL/6 background, deficient in antibody secretion, immunized and challenged as in (A). AID^*-/-*^ mice do not show significant protection, indicating the importance of antibodies. D) Passive transfer of immune sera confers protection against a lethal blood stage challenge. BALB/cJ mice received iv injections of 300μL serum from 3 x 50,000 *Pyfabb/f* ^*—*^ sporozoite-immunized C57BL/6 mice on days 0, 3 and 5 following a lethal challenge with 10^4^ *Py* iRBCs. Comparisons in (A) and (B) were performed by Student t test where significance is indicated by: *0.05≥p>0.01

To assess the relative importance of antibodies and T cells in protection, BALB/cJ and C57BL/6 mice were immunized with LAGAP sporozoites, isolated from mosquito salivary glands, and given an intravenous (iv) challenge of 10,000 *Py* lethal strain iRBCs 25 days after the final immunization. While mock-immunized mice (mice injected with uninfected mosquito salivary gland debris) succumbed to hyperparasitemia within a week after challenge, LAGAP-immunized mice of both strains controlled parasitemia and cleared infection (Fig 1A). Interestingly, C57BL/6 immunized mice controlled the BS infection more robustly than BALB/cJ mice, displaying a lower peak parasitemia (∼2% vs. ∼13% at day 8 after challenge Fig 1A). To examine the respective roles of antibodies (Ab) and T cells in protection, we depleted T cells using monoclonal Ab (mAb) specific for CD4 and CD8 24 hours prior to lethal iRBC challenge (S1A and S1B Fig). Strikingly, only LAGAP-immunized C57BL/6 mice were able to control BS infection in the absence of T cells whereas BALB/cJ mice succumbed to hyperparasitemia similar to mock-immunized control mice (Fig 1C). Thus, the immune mechanisms of STP depend on the mouse genetic background. Whereas humoral immunity is sufficient to protect C57BL/6 mice from a lethal BS challenge, T cells are required to protect LAGAP-immunized BALB/cJ mice. Given the stage-transcending immunity observed, we wanted to ensure that there was no self-limiting blood stage infection caused by breakthrough during LAGAP PE stage immunization, which could be inducing blood stage immunity. To do this, 250μL of pooled blood from C57BL/6 mice immunized with LAGAP sporozoites three days prior was injected into naïve C57BL/6 mice. None of the 5 recipient mice became blood stage patent. This contrasts with transfer of just 2 LAGAP blood stage parasites, which can result in patency of 100% of mice by day 7[29]. Thus, LAGAP PE immunization did not induce a submicroscopic blood stage infection that could be causing the observed blood stage immunity.

To further examine if antibodies elicited by LAGAP immunization are required for protection against BS infection, we immunized C57BL/6 AID^-/-^ mice, which possess B cells that are incapable of producing class-switched antibodies [30,31]. These mice developed a robust CD4^+^ and CD8^+^ T cell response to immunization as measured by markers of antigen-experienced cells in the peripheral blood[32] (S2A and S2B Fig) but failed to control a lethal BS challenge (Fig 1C). Furthermore, passive transfer of wildtype C57BL/6 immune sera to BALB/cJ mice conferred protection against a lethal BS challenge in all mice (Fig 1D). This confirms the ability of antibodies raised in C57BL/6 LAGAP-immunized mice to control BS infection and demonstrates that the difference in protection between the two strains is not explained by a higher susceptibility to infection in BALB/cJ mice. Thus, these data indicate that the antibodies elicited by LAGAP immunization of C57BL/6 mice are potent and essential for STP against BS infection.

**Fig 2:**
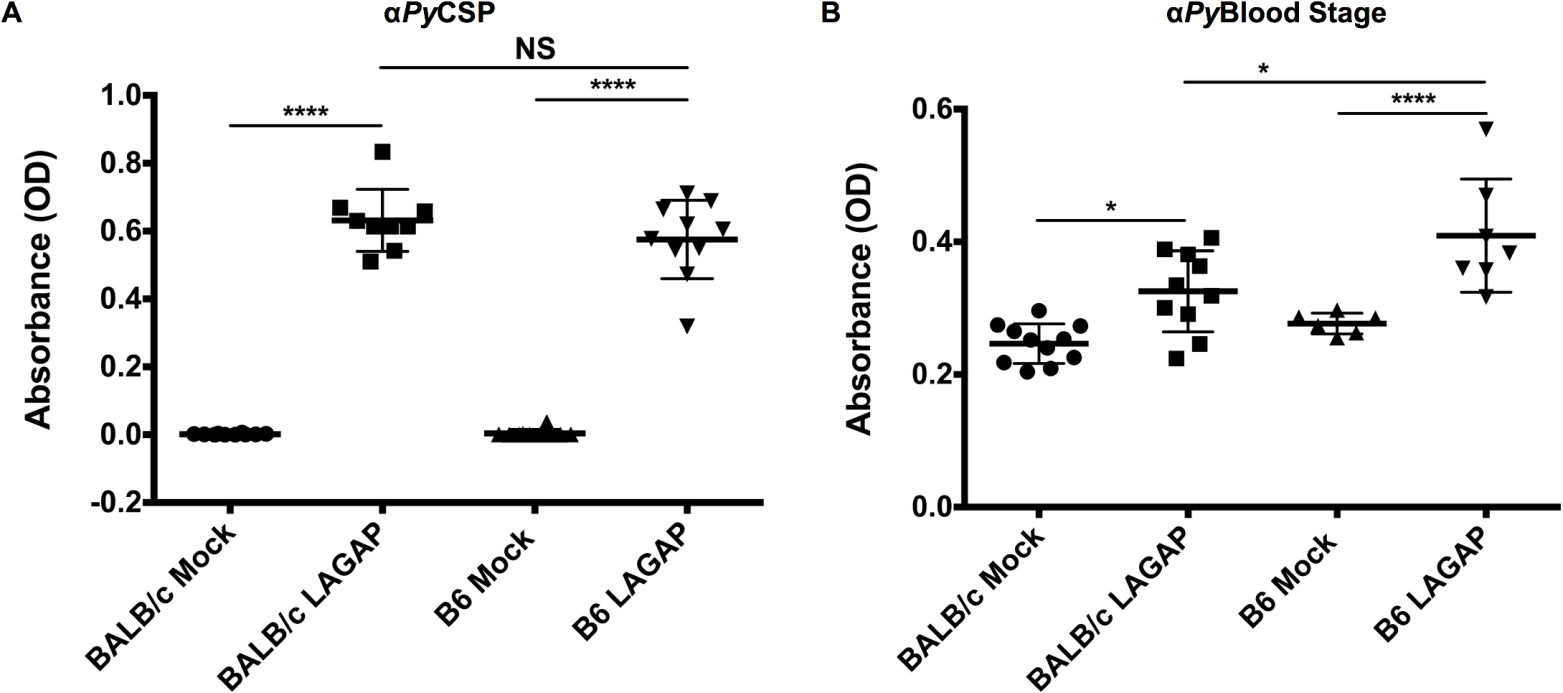
Immunization of C57BL/6 and BALB/cJ mice with LAGAP elicits antibodies against both sporozoites and BS parasites. Serum from BALB/cJ and C57BL/6 mice (n = 10 mice per group, over two independent immunizations) immunized with 3 x 50,000 *Pyfabb/f-* sporozoites was collected 2 weeks after the final immunization and used in ELISA to detect total IgG against CSP (A) and blood stage lysate (B). C57BL/6 and BALB/cJ mice produce antibodies against both CSP and BS proteins with higher anti-BS titers in C57BL/6 mice. Comparisons were performed using one-way ANOVA with Tukey post-hoc analysis with significance indicated by: *0.05≥p>0.01; **0.01≥p>0.001; ***0.001≥p>0.0001; ****p≤0.0001; non-significant (NS) p≥0.05.

To further investigate the host strain-specific differences in STP, we characterized the titers of IgG antibodies against sporozoites and BSs in sera obtained from LAGAP-immunized mice of both strains. Both C57BL/6 and BALB/cJ had similar IgG responses to CSP after immunization (Fig 2A), indicating that humoral immune responses to the major sporozoite surface protein are similar in both strains. Although both strains showed an increase in total IgG against BS parasites following LAGAP immunization, C57BL/6 mice had significantly higher titers than BALB/cJ mice (Fig 2B).

Taken together, these data show that both cell-mediated and humoral immunity protects against BS infection following LAGAP immunization. Furthermore, antibodies from LAGAP immunized C57BL/6 but not BALB/cJ mice are both sufficient and essential for STP. The observation that immunization of C57BL/6 mice produces higher antibody titers against BS proteins when compared to immunization of BALB/cJ mice might contribute to the superior protection against a lethal BS challenge observed in the former.

### Multiple antibody effector mechanisms are required for STP in LAGAP-immunized C57BL/6 mice

**Fig 3:**
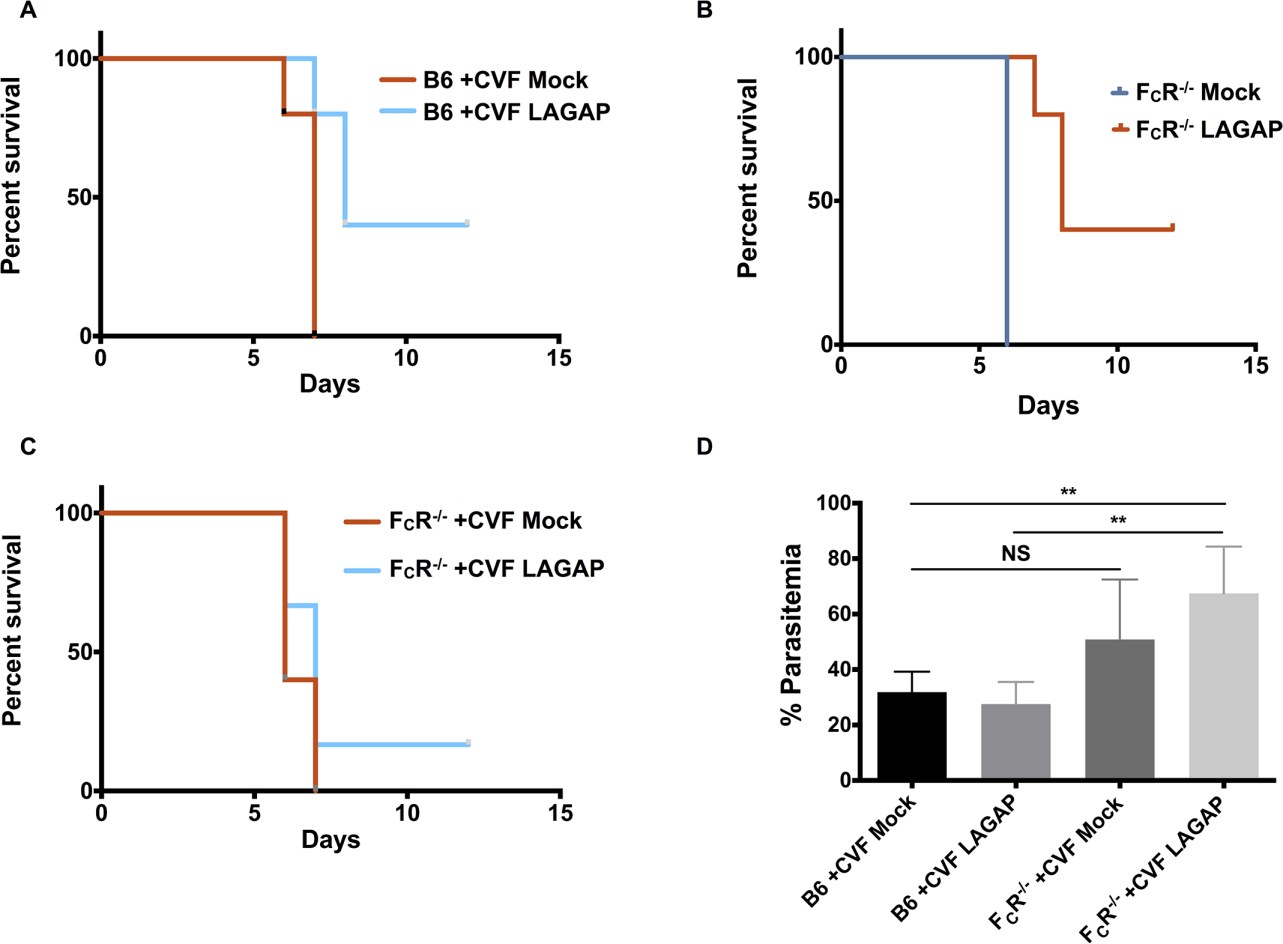
Antibodies elicited by LAGAP immunization require complement and F_C_R binding for complete protection. A) Survival of C57BL/6 mice (n = 5 mice per group) immunized and depleted of CD4 and CD8 T cells as in Fig 1. Six hours prior to challenge with 10^4^ iRBC, mice were depleted of complement by administration of 30 μg of cobra venom factor with an additional dose at 4 days post challenge. Loss of protection in 3/5 mice following complement depletion indicates a strong role for the classical complement pathway in antibody-mediated BS protection. B) Survival of FcγR^*-/-*^ given the same immunization, T cell depletion and challenge as in Fig 1 without complement depletion by CVF. Loss of protection from lethal parasitemia also implicates F_C_R binding in antibody-mediated BS protection. C) The same immunization and challenge was performed with FcγR^*-/-*^ mice but with complement depletion prior to challenge as in (A). Loss of protection in 4/5 mice further confirms the role of both complement and F_C_R binding as antibody effector mechanisms. D) Parasitemia of mice in (A) and (C) on day 5 post challenge. A higher peak parasitemia in complement-depleted FcγR^*-/-*^ mice confirms the role of F_C_R-binding in controlling parasitemia in LAGAP-immunized mice. Comparisons were performed using one-way ANOVA with Tukey post-hoc analysis with significance indicated by: **0.01≥p>0.001; non-significant (NS) p≥0.05.

Antibodies can function independent or dependent of the F_C_ portion of antibodies. F_C_-independent mechanisms include interference with pathogen activities by steric hindrance or blocking of target proteins (e.g. pathogen ligands for host cell infection). The F_C_-dependent mechanisms include complement-mediated lysis of the target pathogen and opsonization of the pathogen or pathogen-infected cell, flagging it for phagocytosis or destruction by F_C_-receptor (F_C_R)-bearing cells. To determine which mechanisms were playing a role in the antibody-mediated STP observed, we immunized C57BL/6 mice with LAGAP as before, depleted them of T cells and then additionally depleted complement via injection of cobra venom factor (CVF). CVF is a C3 convertase, which rapidly and efficiently depletes complement within hours of administration for 3–5 days[33] (S3A Fig). We injected 30 μg of CVF 6 hours prior to challenge and 4 days after challenge to ensure complement depletion throughout BS challenge. When LAGAP-immunized C57BL/6 mice were depleted of complement and T cells, only 40% survived a lethal blood stage challenge (Fig 3A). In contrast, 100% of immunized C57BL/6 mice lacking T cells but not depleted of complement survived the same challenge (Fig 1B). This indicates a strong role for complement-mediated destruction of opsonized parasites and/or iRBCs in the elimination of a BS infection in immunized mice. We also performed similar immunizations with C57BL/6 F_C_Rγ^-/-^ mice—which lack the γ-chain subunit of the FcγRI, FcγRIII and FcεRI receptors—to determine the role of F_C_-receptor binding in protection. These mice developed antibody titers against sporozoites and BS parasites that were comparable to wild type C57BL/6 mice (S3B and S3C Fig). Yet, lack of F_C_R functions also resulted in a reduction of mouse survival after lethal BS challenge from 100% (Fig 1B) to 40% (Fig 3B), implicating this effector pathway in LAGAP-elicited antibody-mediated protection. Elimination of all F_C_-dependent effector functions by CVF administration in immunized F_C_Rγ^-/-^ mice further reduced survival to 20% (Fig 3C and 3D). This again indicates a strong role for F_C_-dependent antibody effector mechanisms in LAGAP-immunized mice. The survival of a small proportion of mice suggests that F_C_-independent basic neutralization of parasites by LAGAP-elicited antibodies is also contributing to protection, although this was minor when compared to F_C_-dependent protection.

### Antibodies induced by immunization with LAGAP recognize PE stage- and BS parasites

**Fig 4:**
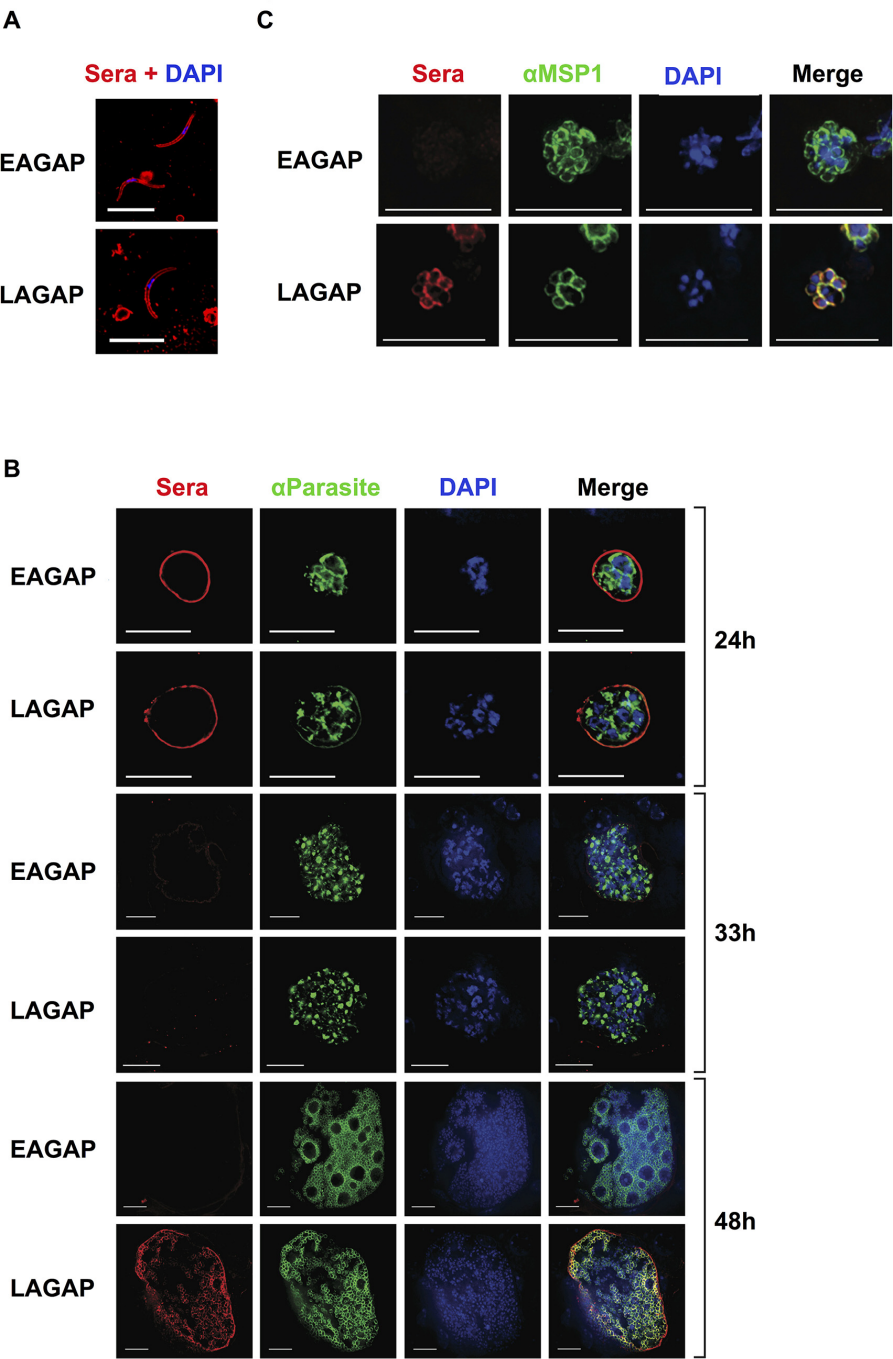
Antibodies elicited by a late liver stage-arresting but not early liver stage-arresting GAP recognize antigens of both late liver stages and blood stages. C57BL/6 mice were immunized twice with 50,000 LAGAP or EAGAP sporozoites. Serum was collected prior to immunization (“pre-immune”) and two weeks after the last immunization for use in immunofluorescent assays (IFA) against parasite life cycle stages. IFAs were performed using fixed salivary gland sporozoites (A), liver sections of infected mice obtained at 12, 24, 33 and 48 hours post-infection (B) and blood stages (C). Parasites were also visualized using antibodies recognizing binding immunoglobulin protein (BiP) and MSP1 (for 48h liver stages and BSs). DNA was visualized with 4',6-diamidino-2-phenylindole (DAPI). These data indicate that antibodies in LAGAP-immunized C57BL/6 mice recognize all parasite life stages whereas EAGAP immune serum only recognizes sporozoites/early liver stages. Scale bar: 10 μm.

LAGAP elicit STP and antibodies play a pronounced role in this protective immunity. It has been shown previously that RAS and early liver stage-arresting GAP (EAGAP) do not elicit STP[14,34]. Thus, we predicted that the antibodies mediating STP are elicited by antigens expressed in late liver stage parasites and that these antigens are shared with BS parasites. To analyze the targets of STP, we investigated the stages of the parasite that are recognized by LAGAP-elicited antibodies using immunofluorescence assay (IFA). As a control, we used serum collected from C57BL/6 mice immunized with the EAGAP, *Pysap1* ^*-*^, which efficiently invades hepatocytes but is completely attenuated by 6h post infection[35]. Antibodies from both EAGAP and LAGAP-immunized mice recognized sporozoites with a circumferential surface-staining pattern, likely indicative of CSP recognition (Fig 4A). Staining of liver stage parasites 24h post-infection with the same immune sera also showed a circumferential pattern for both sera (Fig 4B). Interestingly, both immune sera showed little/no reactivity against 33h-old liver stage parasites (Fig 4B). However, we observed pronounced differences in reactivity against 48h late liver stage parasites, a time when exoerythrocytic merozoites begin to differentiate. While there was little/no detectable reactivity with EAGAP immune serum (Fig 4B), LAGAP immune serum showed robust reactivity that localized to the exoerythrocytic merozoite surface and to the parasitophorous vacuole membrane (Fig 4B). We next performed IFA with immune sera on BS parasites to determine if the antibodies cross-reacted with these stages. While EAGAP immune serum had no detectable reactivity, LAGAP immune serum displayed an intense circumferential staining on merozoites that co-localized with MSP1 (Fig 4B). Interestingly however, we did not detect antibodies against either the 19 or 42kD fragment of merozoite surface protein 1 (MSP1) in LAGAP-immunized mice (S4 Fig). This provides an unprecedented demonstration that LAGAP immunization elicits antibodies against the late liver stages and BSs, which are mostly reactive with merozoite surface determinants. This could constitute one major mechanism by which STP is achieved.

### Anti-BS antibodies elicited by LAGAP immunization in BALB/c and C57BL/6 mice differ in specificity

**Fig 5:**
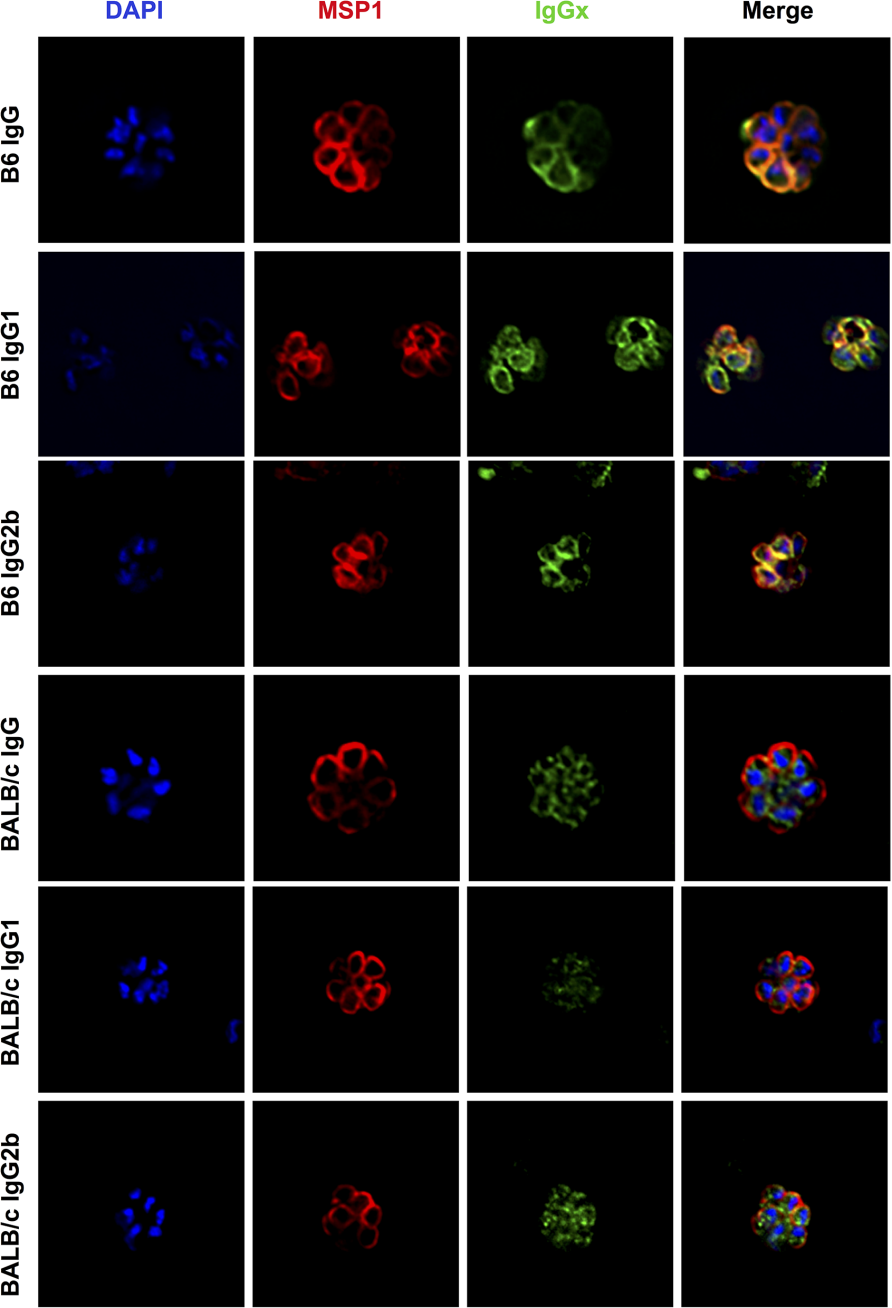
Antibodies elicited by LAGAP in C57BL/6 but not BALB/cJ mice are of broad isotypes and recognize the merozoite surface. Serum was collected from LAGAP immunized C57BL/6 (top 3 rows) and BALB/cJ (bottom 3 rows) mice as in Fig 4 and used in a blood stage IFA. Secondary antibodies against total IgG, IgG1 and IgG2b were used to visualize isotype-specific anti-blood stage antibodies. Parasites were also visualized with antibodies recognizing MSP1 and DNA visualized with 4',6-diamidino-2-phenylindole (DAPI). Antibodies in LAGAP-immunized C57BL/6 and BALB/cJ mice do not differ in isotype but rather in antigen specificity with only C57BL/6 serum recognizing the merozoite surface.

Both LAGAP immunized BALB/cJ and C57BL/6 immunized mice produce antibodies that can recognize BS proteins by ELISA with C57BL/6 producing slightly higher titers (Fig 2B). This quantitative difference, however, cannot explain the inferior protection afforded by antibodies in BALB/cJ as passive transfer of C57BL/6 immune serum to naive BALB/cJ mice results in antibody titers as low as actively immunized BALB/cJ mice (S5 Fig), yet these passively immunized BALB/cJ mice are still protected against a BS challenge (Fig 1D). Thus, the differential protection could be due to different BS antigens being recognized by antibodies from the two strains or, given the demonstrated role of F_C_-mediated functions, by differences in the isotype distribution of the antibodies. To determine if the antibodies produced by the two strains of mice differ qualitatively by either specificity or isotype, we performed IFAs on iRBCs using serum from both LAGAP-immunized BALB/c and C57BL/6 immunized mice and secondary antibodies representing different IgG isotypes. Immunized C57BL/6 mice produced IgG of both IgG1 and 2b isotypes which co-localized with MSP1 at the surface of exoerythrocytic merozoites (Fig 5). In contrast, IFAs using serum from LAGAP-immunized BALB/cJ mice showed antibodies that are primarily of the IgG2b isotype and recognized the parasite interior (Fig 5). Quantification of immune serum staining patterns in 65 iRBCs confirmed the dichotomy of C57BL/6 serum recognizing the periphery of schizonts, whereas BALB/c immune serum recognized the parasite interior (Table 1). Western blots probing BS lysates with immune sera also demonstrated a distinct set of proteins recognized by sera from C57BL/6 immunized mice that were not apparent in serum from BALB/cJ immunized mice (S6 Fig). Thus, although immunized BALB/cJ mice produce anti-BS antibodies that were detectable by ELISA, IFA and Western blot, these antibodies offered no protection against a lethal BS challenge (Fig 1C). Therefore, whereas LAGAP-immunized BALB/cJ mice produce non-protective anti-BS antibodies, LAGAP-immunized C57BL/6 mice produce antibodies that recognize a unique set of BS antigens capable of potent STP.

**Table 1:** Quantification of staining patterns of sera from LAGAP-immunized mice.

Mouse Strain	Interior Staining	Peripheral Staining	Both
**C57BL/6**	0	58	7
**BALB/cJ**	62	0	3

## Discussion

LAGAP are unique amongst all current malaria immunization strategies in that they are designed to arrest the immunizing parasites late in liver stage development, cause no exposure to BS parasites and yet protect against PE parasite- and BS parasite challenge [28]. Here, we show that STP is mediated by T cells and antibodies, with that the latter recognizing antigens shared between the late liver stage and BS parasites. Immunization strategies focusing on single stages of infection must either be 100% effective in preventing PE infection of the liver or they must overcome the significant antigenic diversity, immune evasion mechanisms and high parasite burden present during BS infection in order to prevent disease and transmission. Other whole sporozoite vaccination types such as RAS or EAGAP provide potent, antigenically diverse PE immunity but they require complete prevention of development of even a single liver stage parasite. Otherwise they would fail to confer protection. In contrast, LAGAPs that elicit STP can maintain efficacy in the face of potentially leaky PE protection and breakthrough BS infection if one or a few parasites escapes PE immunity. Here, we demonstrate for the first time that in addition to PE immunity, immunization with LAGAP invokes both protective cellular and humoral BS immune responses. This not only provides a platform for investigation of novel cross-protective antigens and immune mechanisms, but together with the robust PE immunity observed after LAGAP immunization[24] provides further rationale for development of LAGAP for potential use in human immunization.

It has been previously shown that immunization with LAGAP elicits both robust cellular and humoral PE immunity[24,28]. Thus, it was reasonable to hypothesize that the observed STP against a BS challenge in LAGAP-immunized mice could be mediated by antibodies, CD4^+^ or CD8^+^ cells, as all have also been implicated in controlling BS infection[36–41]. Our data demonstrate that immunization with LAGAP elicits functional T cell responses to BS parasites that are essential for protection in BALB/cJ mice. Further studies using antibody-deficient mice on the BALB/cJ background would be required to determine which cell types are involved and if these cells are sufficient for protection in the absence of antibodies. Conversely, immunized AID^-/-^ mice on the C57BL/6 background were unable to control a lethal BS challenge, pointing to antibodies as critical for protection. However, their parasitemia was curtailed and their time to death longer than WT controls, indicating a role for effector T cell immunity in this strain as well. CD8 T cells are widely recognized as essential effectors in eliminating liver stage parasites[8], and their role in BS protection is becoming more evident[38,39,41]. Although data demonstrating a clear role for CD8 T cells in mediating blood stage immunity in humans is lacking, identification of the antigens recognized by CD8 T cells in LAGAP-immunized BALB/cJ mice might be useful as these antigens are potentially present in multiple stages, are protective targets and thus could be prime candidates for a cross-stage protective T cell subunit vaccine.

In contrast to the increasingly appreciated role of T cells in BS immunity, antibodies have long been considered the main mechanism of protection against BS parasitemia and disease. This is based on early studies showing that passive transfer of convalescent serum from malaria-experienced individuals to unprotected individuals resulted in protection against BS disease [42,43] and high antibody titers against BS antigens correlate with reduction of morbidity and mortality in endemic areas [43,44]. However, whether or not this is mediated simply by antibody binding and impairment of merozoite activities, such as invasion, or mediated also by F_C_-dependent effector mechanisms still remains unclear. One study in mice using passive transfer of *Py* hyperimmune sera or an anti-MSP1 mAb to wildtype and F_C_Rγ^-/-^ mice suggested that F_C_-mediated mechanisms are dispensable[45]. However, additional studies in animal models and naturally immune individuals highlighted the importance of “cytophilic” antibodies (IgG1 and IgG3 in humans, IgG2a/b in mice) acting through F_C_-dependent functions for control of BS parasitemia[46–54]. In our current study, the antibodies engendered by LAGAP immunization are strongly dependent on F_C_R binding as the majority of immunized F_C_Rγ^-/-^ mice lost protection despite high levels of antibodies. F_C_-mediated complement fixation and destruction of antibody-bound iRBCs, merozoites or parasite proteins in immune complexes (the “classical complement pathway”) has been poorly defined. Only a few *in vitro* studies[55–57] have implicated complement fixation in the destruction of parasites while the one *in vivo* study conducted in non-human primates concluded that complement depletion via CVF had no impact on natural control of parasitemia[58,59]. In contrast, opsonized-iRBC phagocytosis by macrophages has been well documented and has been correlated with protection in naturally immune individuals[52,60]. Our data suggest a strong role for the classical complement pathway as immunized mice lacking complement showed a severe defect in controlling BS infection in the presence of LAGAP-induced antibodies. Importantly, localization data indicate that the antibodies do not preferentially bind the surface of iRBC but strongly react with the merozoite surface, suggesting that these antibodies bind and fix complement directly on the merozoite. This is in line with a previous study demonstrating that antibodies recognizing the merozoite protein SERA have enhanced inhibitory capacity in the presence of complement *in vitro*[57]. Complement fixation by opsonized merozoites could enhance parasite clearance by a number of mechanisms including direct killing via the membrane attack complex, recruitment of leukocytes via generation of anaphylatoxins (C3a and C5a) or by opsonization and subsequent phagocytosis of complement-bound parasites. Regardless of the mechanism, our data provide the first *in vivo* evidence of the functional importance of the classical complement pathway playing a major role in the control of blood stage parasitemia.

We have previously speculated that the STP resulting from LAGAP immunization is targeting protective antigens that are shared between the late liver stages and BS parasites[28]. This arises from the observation that parasites arresting development early in the liver, such as RAS or EAGAP, do not afford STP[15,34]. Here, we provide direct evidence that it is indeed antigens shared between the late liver stages and BSs that are the targets of protection. IFAs using serum from mice immunized with an EAGAP (*Pysap1* ^*-*^,) and the LAGAP show that immunization with both parasites elicits antibodies against sporozoites and liver stages up to 24 h post infection. This is consistent with the presence of CSP on the sporozoite- and liver stage surface and the abundant anti-CSP antibody titers in the immune sera. In contrast, only LAGAP-immune serum recognized late liver stages, exoerythrocytic merozoites and BS merozoites. Combined with our data showing that LAGAP-induced antibodies alone provide protection from iRBC challenge, this confirms that there are indeed yet to be identified antigens in late liver stage parasites and BS parasites capable of eliciting STP. Targeting these antigens by both T cells and antibodies (i.e. with viral vectors) could allow for multiple opportunities to eliminate the parasite in both the liver and blood if it is indeed the same antigens providing both PE and blood stage protection. Importantly, this immunity does not appear to target MSP1_19_ or MSP1_42_, which although capable of conferring protection in mice, has failed to protect in clinical studies[26,27].

How exactly LAGAP antigens are acquired and presented by antigen presenting cells (APC) remains to be elucidated. Numerous types of cells in the liver are capable of antigen uptake and presentation including Kupffer cells (KC, liver-resident macrophages), multiple types of dendritic cells (DC), liver sinusoidal endothelial cells (LSEC) and even hepatocytes[61,62]. Hepatocytes only possess MHCI and can present parasite antigens[63] but lack MHCII and thus this cannot explain the antibody responses to late liver stages we observed. Even though LSEC are very efficient at presentation of exogenous antigen[62], their ability to generate the type of mature, class-switched IgG response seen in LAGAP immunization has not been established[61]. A likely scenario is that as the LAGAP parasite dies late in liver stage development, the hepatocyte undergoes apoptosis[64,65] and releases parasite antigens to liver-resident DC or KC[66], which prime responses to late liver stage/blood stage antigens. Priming against early PE antigens likely occurs against extracellular sporozoites and liver stage parasites that die early as a part of normal parasite infection[29,64,65]. This speculation is bolstered by the fact that we see serum reactivity to early (12 and 24h) and late (48h) liver stages but not to 33h as fewer parasites are dying at this mid liver stage. These liver-resident APCs can then migrate to draining lymph nodes where they prime productive adaptive immune responses. In support of this, a recent report by Lau *et al*. demonstrates that following immunization with RAS by iv injection, substantial parasite-specific T cell activation occurs in the liver-draining lymph nodes at a rate that is surpassed only by the spleen[67]. Another study showed accumulation of CD8α^+^ DCs in the liver of RAS-immunized mice and that these DCs were capable of directly activating T cells *in vitro*[68]. Several reports identify peripheral lymphoid organs such as the spleen as instrumental in immune priming following sporozoite immunization[67,69], but direct evidence of peripherally primed T cells being the mediators of protection after whole sporozoite immunization by iv injection is lacking. As the antigens mediating STP in our model are present in late liver stages, it is more likely that liver-resident APCs are responsible for priming the immune response to late liver antigens in the liver-draining lymph nodes.

Antibodies in LAGAP immune sera overwhelmingly recognize the surface of the developing and mature merozoite and co-localize with MSP1 in late liver stage and BS parasites. MSP1 is the most abundant protein on the merozoite surface and antibodies against this protein have been shown to be protective against BS infection in mice [70–73]. However, we were unable to detect antibodies against either protective MSP1 regions in serum from LAGAP-immunized mice[74,75]. Thus, the protective antigen(s) are as yet unidentified merozoite surface protein(s). Our data from IFAs and Western blot indicate that the protective antibodies in LAGAP-immunized C57BL/6 mice recognize different antigens than non-protective antibodies from BALB/c immunized mice. Accordingly, by identifying the antigens uniquely recognized by C57BL/6 immune serum, it is conceivable that a subset of these antigens could be incorporated into a multi subunit vaccine that could induce STP.

The potent STP observed in our studies also lends support to using LAGAP as a whole parasite vaccination strategy. The only other example of true STP with live parasites has been observed in mice immunized with iRBC under chloroquine cover[76]. This immunization strategy in mice controlled liver infection via CD4^+^ and CD8^+^ T cells, conferred partial BS immunity and has been demonstrated as protective against sporozoite challenge in humans[77]. Multi-stage protection has also been demonstrated in animal models of ITI (infection with wild type sporozoites under drug cover) where there is both antibody-dependent and independent protection against both a sporozoite and BS infection[25,76,78]. However, this is not truly stage transcending protection as the development of BS immunity requires exposure to low levels of BS parasitemia during immunization[25,78].

Trials using cryopreserved RAS in humans have demonstrated that administration of live, attenuated sporozoites is effective, safe and well-tolerated[79]. Significant hurdles remain in manufacturing and delivering a live, attenuated sporozoite vaccine, but the success of RAS in humans has provided the impetus for creative solutions to these barriers. Yet, no gene knockout in the human-infective *P*. *falciparum* species has been created that is phenotypically similar to the LAGAP described here and knockout of the orthologous *P*. *falciparum* gene results in a parasite incapable of forming sporozoites[80]. Given the promise of superior and stage-transcending immunity, development of a late liver-arresting *P*. *falciparum* GAP that is free from breakthrough during immunization is of high priority and should be under intense investigation.

In summary, we have shown that immunization with LAGAP can elicit both T cell and antibody-mediated immunity to BS parasites via recognition of antigens shared between the late liver stage- and BS parasites. Furthermore, antibodies act through complement and F_C_R binding to control and eliminate BS parasitemia. Since these T cells and antibodies are both highly efficacious and directed against potentially novel antigens, mechanistic studies using this model can critically inform the development of the next generation of subunit vaccines. These should be designed to elicit T cells as well as antibodies of the correct isotype, each directed against critical antigens and effective in eliminating liver stages and blood stage parasites.

## Materials and Methods

### Mice

6–8 week old female BALB/cJ and C57BL/6 mice were purchased from the Jackson Laboratory. Age-matched female F_C_Rγ^-/-^ mice on the C57BL/6 (B6.129P2-*Fcer1gtm1Rav* N12, model 583) background were purchased from Taconic Biosciences, Inc. All mice were maintained in a pathogen-free facility accredited by the Association for Assessment and Accreditation of Laboratory Animal Care at the Seattle Biomedical Research Institute. All experiments were conducted in accordance with animal protocols approved by the Institutional Animal Care and Use Committee.

### Parasite growth and sporozoite isolation

Six-to-eight week old female SW mice were injected with blood from *Py* knockout (*fabb/f-* or *sap1-*)-infected mice to begin the growth cycle. The infected mice were used to feed female *Anopheles stephensi* mosquitoes after gametocyte exflagellation was observed. On days 14–17 post infectious blood meal, salivary gland sporozoites were isolated from the mosquitoes for experimentation.

### Mouse immunizations

Mice were immunized by injecting 50,000 sporozoites intravenously via the tail vein two weeks apart. As a control, equivalent amounts of salivary gland debris from uninfected mosquitos were used.

### BS challenge

Frozen blood stocks of *Py*XL or *Py*YM-infected blood containing 1% iRBCs was ip-injected into BALB/c or C57BL/6 mice and allowed to develop for 2–4 days until parasitemia reached a maximum of 1% as determined by Giemsa-stained thin smear. These mice were terminally bled via cardiac puncture and the blood diluted in PBS to contain 10,000 iRBCs/200μL. iRBCs were then iv-injected at a volume of 200μL/mouse into congenic recipient mice. Parasitemia was monitored by Giemsa-stained thin smears beginning on day 3 post-infection. Mice were euthanized when parasitemia reached 60% or became moribund.

### Passive transfer

Serum from mice immunized with three doses of *Pyfabb/f* ^*-*^ sporozoites or uninfected salivary gland debris (mock) was collected on day 7 and day 14 after the final immunization and pooled. Naïve mice were intravenously injected with 300 μl of pooled serum on day 0, 3 and 5 after a challenge with 10,000 lethal PyXL or PyYM iRBCs injected intravenously.

### CD8^+^/CD4^+^ T cell depletion

CD8^+^ and CD4^+^ T cells were depleted in mice as previously described [22]. Briefly, 0.5 mg of anti-CD8 mAb 2.43 (BioXCell) and 0.35 mg of anti-CD4 mAb 1.5 (BioXCell), or 0.85 mg of isotype control rat IgG2b (BioXCell) was iv-injected into mice 24 hours prior to parasite challenge. T cell depletion was confirmed before each challenge by collecting 50–100 μl of peripheral blood via the retro-orbital plexus from each mouse and assaying peripheral blood lymphocytes by flow cytometry staining for CD19, CD3, CD4 and CD8.

### Complement depletion with cobra venom factor

Cobra venom factor (CVF) is a complement activating C3b analog that when administered rapidly depletes complement (Vandenberg 1991). For complement depletion, mice were administered 30 μg of CVF intraperitoneally 6h prior and 4 days after iRBC challenge. Depletion of complement was confirmed prior to challenge by C3 sandwich ELISA (Genway Biotech).

### ELISA

Serum was isolated from peripheral blood at day 25 post-immunization, immediately prior to challenge as previously described[24]. ELISA plates (Corning, Inc.) were coated with full length *Py*CSP protein at a concentration of 0.1 μg/mL or with 2 μg/mL of either *Py* sporozoite or BS lysate in calcium bicarbonate/sodium carbonate coating buffer overnight at 4°C. For MSP1 ELISAs, plates were coated at 0.1μg/mL of either the 19 or 42kD fragment (generously provided by Dr. James Burns) as above. Plates were washed prior to addition of a 1:800 (for PyCSP), 1:20 (for sporozoite and BS lysate) or 1:2000 (for MSP1 19 and 42kD) dilution of serum in duplicate followed by incubation at 37°C for two hours. After washing, anti-mouse IgG conjugated to HRP (SouthernBiotech) was added at a 1:2000 dilution for an additional 2h at 37°C. Plates were again washed and 100 μL of SigmaFast OPD (Sigma-Aldrich) substrate was added for 2–10 minutes prior to colorimetric detection of antibodies by measuring absorbance at 450 nm.

### Immunofluorescence analysis

Sporozoites, infected hepatocytes and infected red blood cells were harvested, fixed and stained as previously described [81,82]. Briefly, fixed cells were stained with a 1:200 dilution of serum collected from *Pysap1* ^*-*^ and *Pyfabb/f* ^*-*^ immunized C57BL/6 mice. Rabbit antibodies against BiP and MSP-1 were used as control antibodies for early liver stages and late liver stages/BSs, respectively. Fluorescently labeled secondary antibodies (Alexa Fluor 488 or Alexa Fluor 594) from Life Technologies were used to detect mouse IgG (catalog # A-11059), IgG1 (catalog # A-21125) and IgG2b (catalog #A-21145). Images were acquired using Olympus 1 x 70 Delta Vision deconvolution microscopy. For quantification of staining pattern, slides were prepared as above and blinded to the microscopist. Infected red blood cells containing schizonts were identified by MSP1 staining and anti-mouse IgG staining was classified as “interior”, “exterior” or “both” based on the MSP1 border.

### Statistical analysis

Calculations and statistical tests indicated in the figure legends were performed using GraphPad Prism. A p value < 0.05 was considered significant.

### Ethics statement

All animal procedures were conducted in accordance with and approved by the Seattle BioMed Institutional Animal Care and Use Committee (IACUC) under protocol SK-09. The Seattle Biomed IACUC adheres to the NIH Office of Laboratory Animal Welfare standards (OLAW welfare assurance # A3640-01).

## Supporting Information

S1 Fig Mice were administered anti-CD4 (clone GK1.5) and anti-CD8 (clone 2.43) depleting mAb via ip injection 24 hours prior to challenge. Immediately before challenge, depletion in the peripheral blood was confirmed by flow cytometry staining of cells using mAbs to CD19, CD3, CD4 and CD8. A) Gating strategy for identifying peripheral blood CD4^+^ and CD8^+^ T cells. B) Quantification of T cells as a percentage of lymphocytes after depletion as compared to untreated controls (n = 5 mice per group) shows that T cell depletion is complete and consistent.
(TIF)

S2 Fig Antigen-experienced CD4^+^ T cells (expressed as CD49d^hi^ CD11a^hi^ in A) and CD8^+^ (expressed as CD8α^low^ CD11a^hi^) in B) were identified in the peripheral blood of AID^-/-^ mice 7 days following the indicated number of 50,000 LAGAP sporozoite immunizations. These data show that AID^-/-^ mice are capable of producing robust T cell responses following LAGAP immunization.
(TIF)

S3 Fig A) Mice were administered 30 μg of CVF and serum was collected 6 hours post injection. Complete depletion of complement by CVF was confirmed by ELISA. Serum from F_C_R^-/-^ mice immunized with 3 x 50,000 LAGAP was collected 1 week after the final immunization and used to measure anti-CSP titer in B) as well as anti-sporozoite lysate titer in C) and anti-blood stage schizont lysate titer in D). These data indicate that F_C_R^-/-^ mice are fully capable of producing anti-parasite antibodies at levels comparable to WT mice.
(TIF)

S4 Fig Serum from naïve (n = 4) or 3x LAGAP-immunized (n = 8) C57BL/6 mice was analyzed for anti-MSP1 IgG by ELISA 1 week after final immunization. Serum from a mouse which received 10,000 Py non-lethal infected RBCs and had self-cured was used as a positive control (“B6 Blood Stage”). A difference in OD between naïve and immunized mice was tested by two-way t-test and significance of p<0.05 used as a cutoff. These data confirm that B6 mice immunized with LAGAP fail to make significant anti-MSP1 antibodies to either the 19 or 42kD fragment.
(TIF)

S5 Fig The quantity of anti-blood stage antibodies in BALB/c mice passively immunized with LAGAP-immunized C57BL/6 are equal to that of actively immunized BALB/c mice. Anti-blood stage antibody titer of BALB/c mice iv-injected 3x with 300μL of serum from LAGAP-immunized C57BL/6 mice was measured by ELISA as in Fig 2. Antibody titers are indistinguishable from actively immunized BALB/c mice yet are protective against a lethal blood stage challenge—indicating that antibody quality, not quantity, is responsible for their differential protective capacity.
(TIF)

S6 Fig Blood stage lysate protein was separated on an SDS-PAGE gel and probed with serum from mice of the indicated strain immunized with either 2 x 50,000 EAGAP or LAGAP. In addition, serum from C57BL/6 mice which received a 10,000 iRBC challenge only was also used as a positive control for blood stage antigen exposure. These data further confirm that C57BL/6 and BALB/cJ mice immunized with LAGAP recognize a distinct set of blood stage antigens.
(TIF)
