# Supplementary figures and images for: Mechanisms of Stage-Transcending Protection Following Immunization of Mice with Late Liver Stage-Arresting Genetically Attenuated Malaria Parasites
Source: PLoS Pathog. 2015 May 14;11(5):e1004855. doi: 10.1371/journal.ppat.1004855 (PMC4431720; doi:10.1371/journal.ppat.1004855)

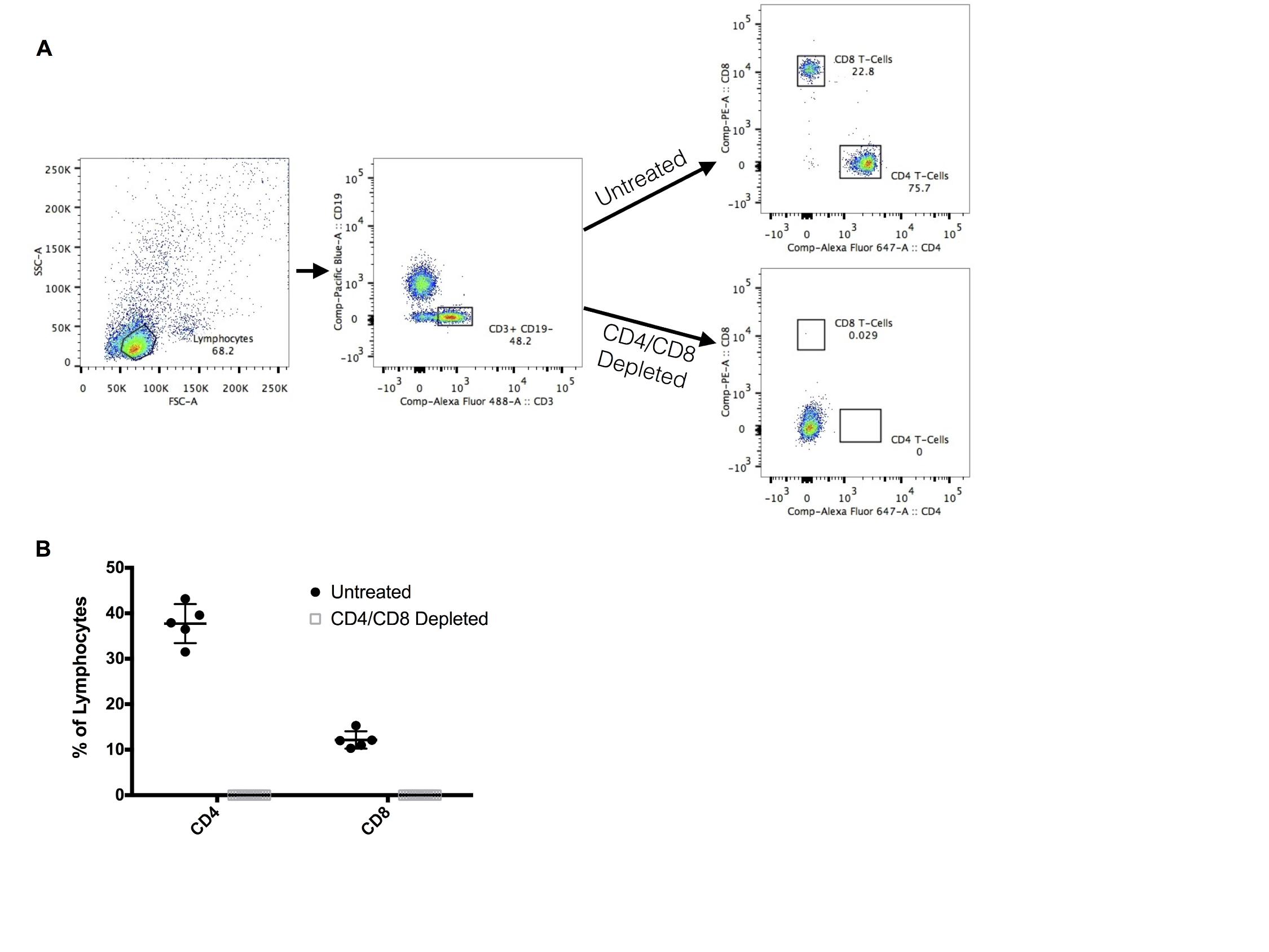

Supplement: S1 Fig — Immediately before challenge, depletion in the peripheral blood was confirmed by flow cytometry staining of cells using mAbs to CD19, CD3, CD4 and CD8. A) Gating strategy for identifying peripheral blood CD4+ and CD8+ T cells. B) Quantification of T cells as a percentage of lymphocytes after depletion as compared to untreated controls (n = 5 mice per group) shows that T cell depletion is complete and consistent. (TIF) [file ppat.1004855.s001.tif]

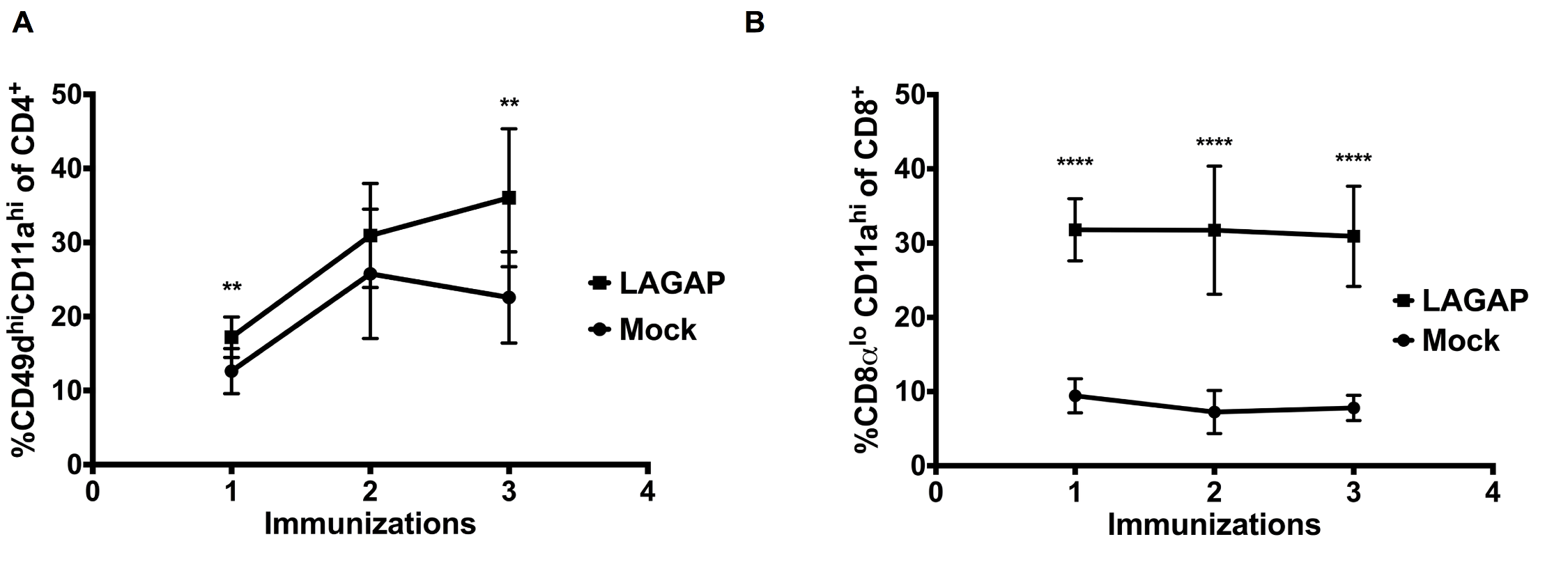

Supplement: S2 Fig — These data show that AID-/- mice are capable of producing robust T cell responses following LAGAP immunization. (TIF) [file ppat.1004855.s002.tif]

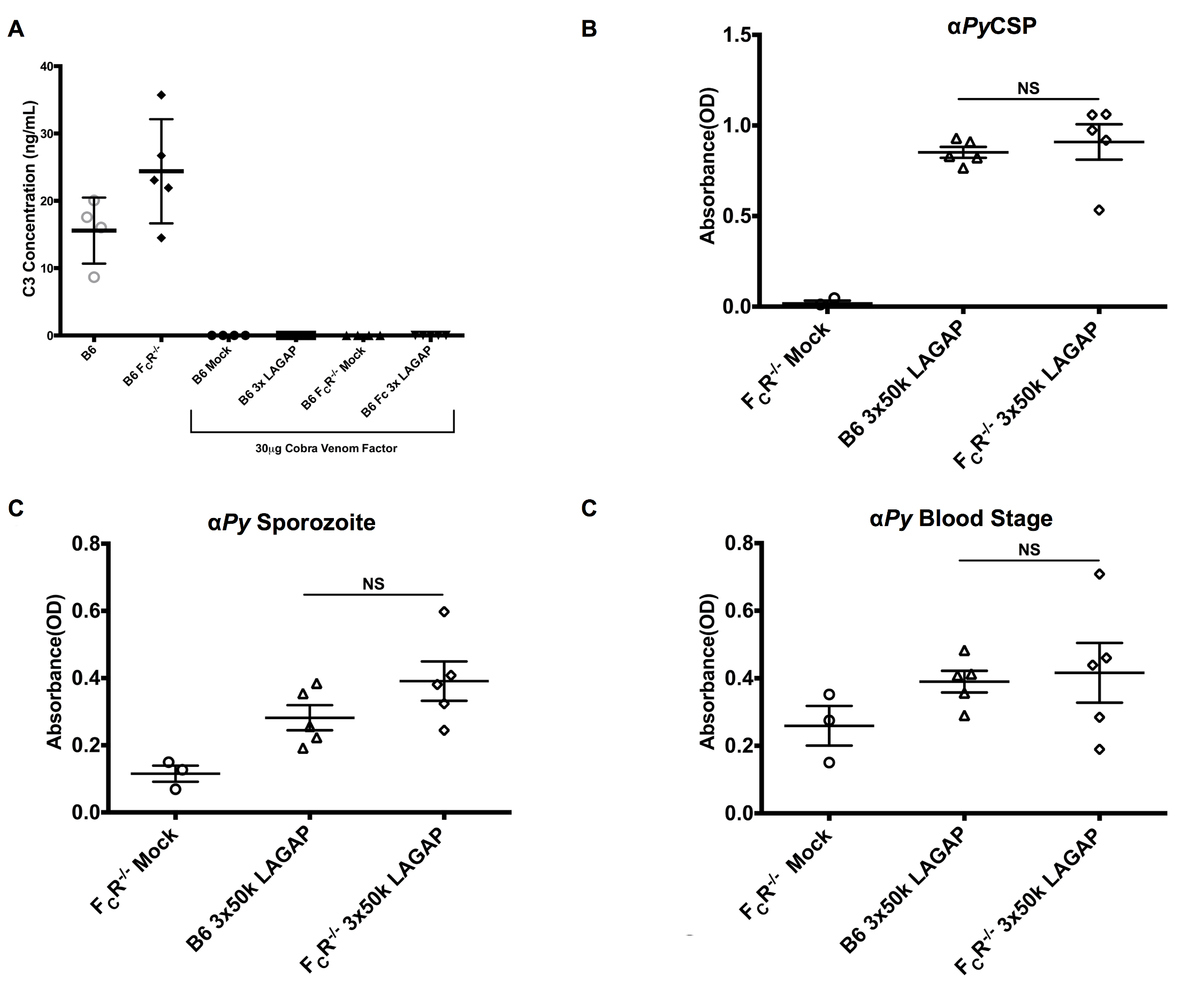

Supplement: S3 Fig — Complete depletion of complement by CVF was confirmed by ELISA. Serum from FCR-/- mice immunized with 3 x 50,000 LAGAP was collected 1 week after the final immunization and used to measure anti-CSP titer in B) as well as anti-sporozoite lysate titer in C) and anti-blood stage schizont lysate titer in D). These data indicate that FCR-/- mice are fully capable of producing anti-parasite antibodies at levels comparable to WT mice. (TIF) [file ppat.1004855.s003.tif]

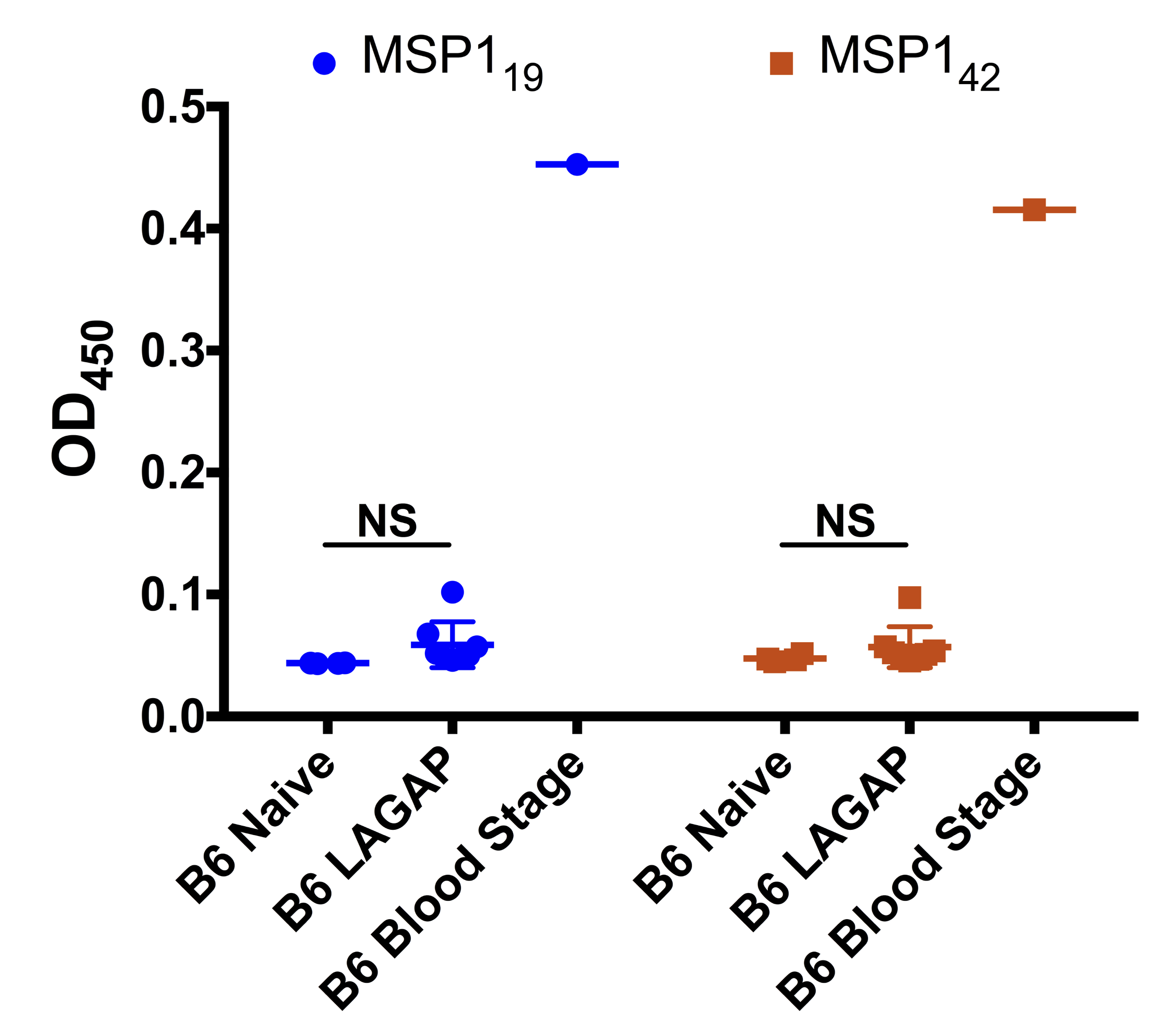

Supplement: S4 Fig — Serum from a mouse which received 10,000 Py non-lethal infected RBCs and had self-cured was used as a positive control (“B6 Blood Stage”). A difference in OD between naïve and immunized mice was tested by two-way t-test and significance of p<0.05 used as a cutoff. These data confirm that B6 mice immunized with LAGAP fail to make significant anti-MSP1 antibodies to either the 19 or 42kD fragment. (TIF) [file ppat.1004855.s004.tif]

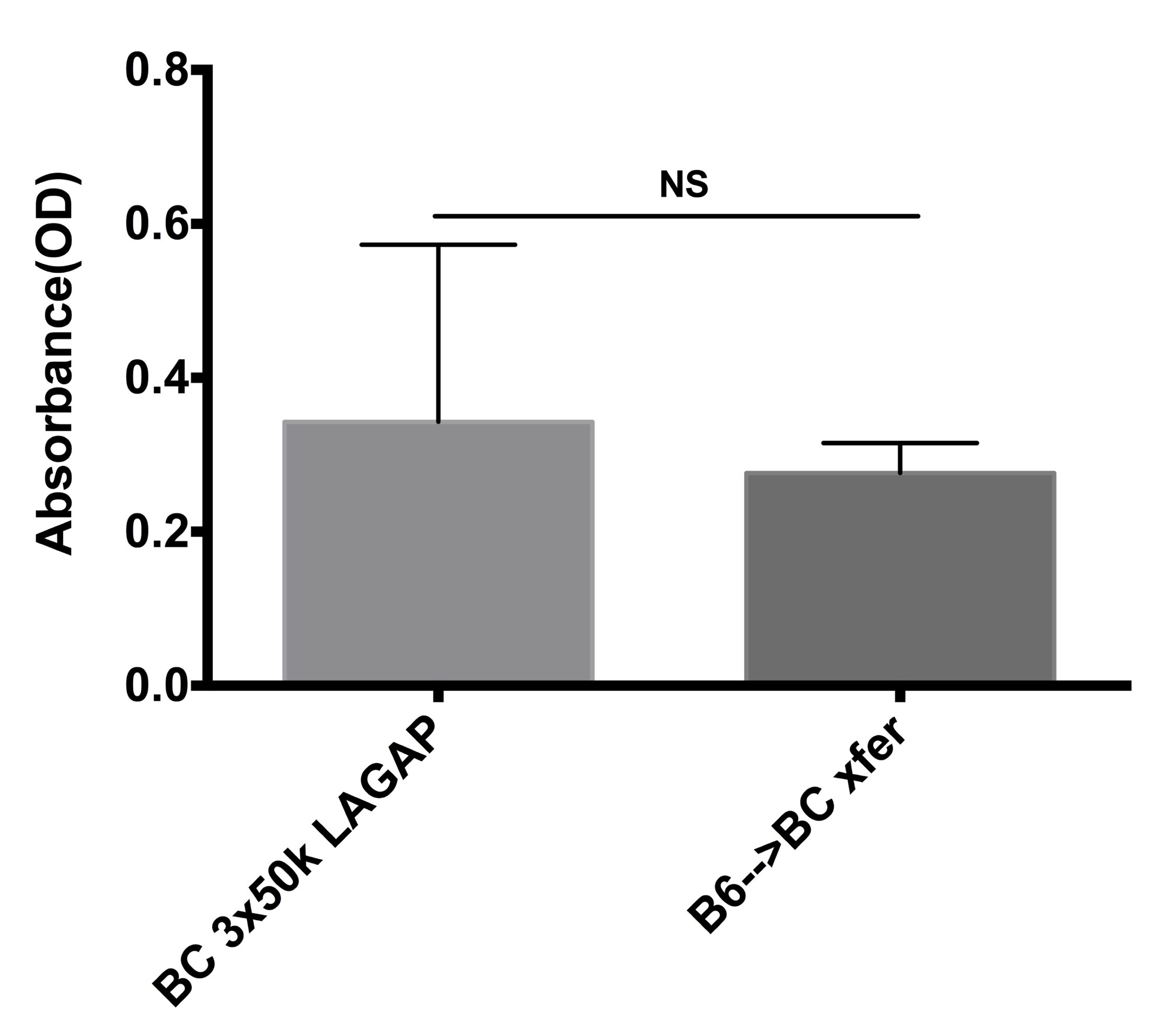

Supplement: S5 Fig — Anti-blood stage antibody titer of BALB/c mice iv-injected 3x with 300μL of serum from LAGAP-immunized C57BL/6 mice was measured by ELISA as in Fig 2. Antibody titers are indistinguishable from actively immunized BALB/c mice yet are protective against a lethal blood stage challenge—indicating that antibody quality, not quantity, is responsible for their differential protective capacity. (TIF) [file ppat.1004855.s005.tif]

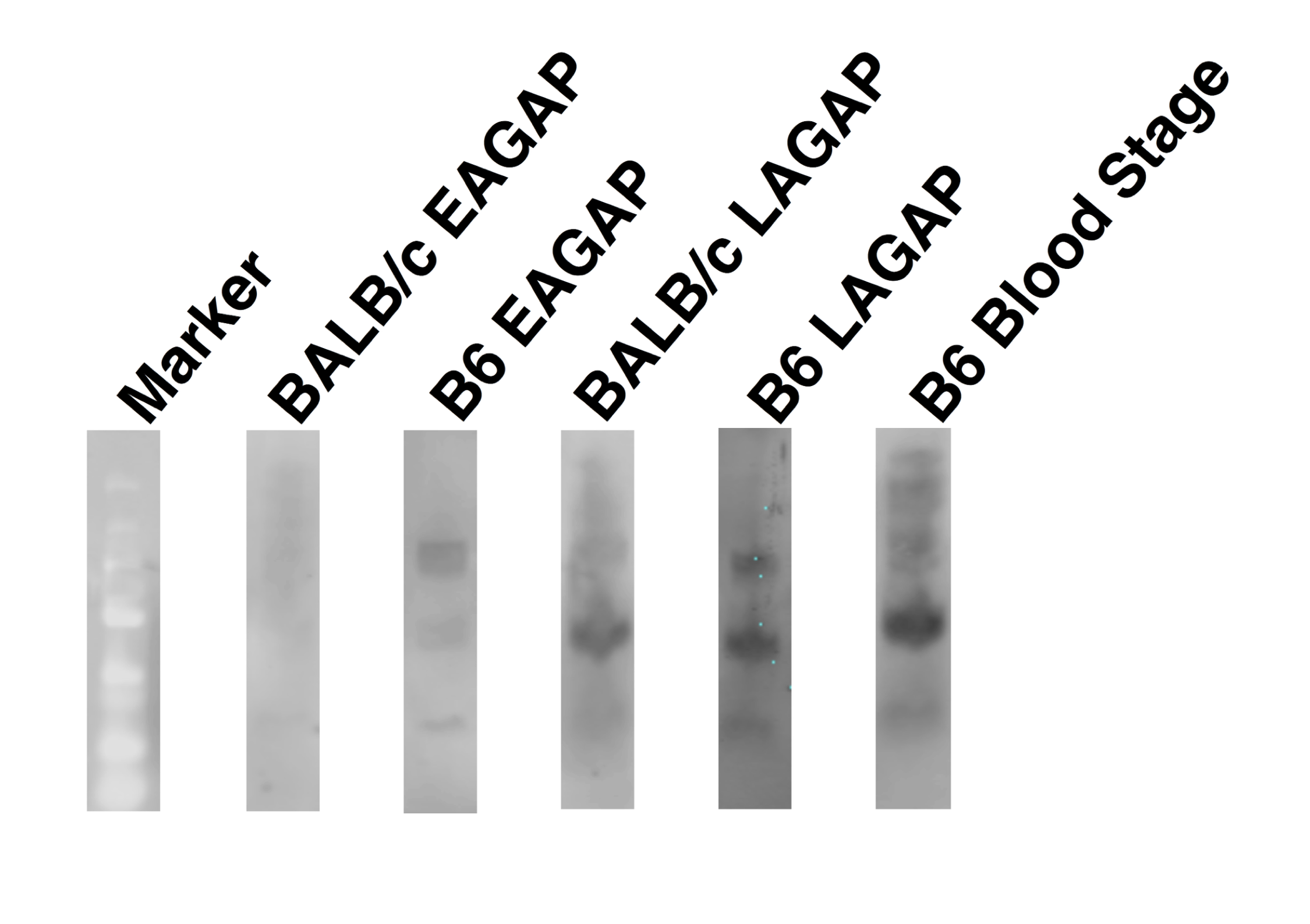

Supplement: S6 Fig — In addition, serum from C57BL/6 mice which received a 10,000 iRBC challenge only was also used as a positive control for blood stage antigen exposure. These data further confirm that C57BL/6 and BALB/cJ mice immunized with LAGAP recognize a distinct set of blood stage antigens. (TIF) [file ppat.1004855.s006.tif]
